# How effective are physical activity interventions when they are scaled-up: a systematic review

**DOI:** 10.1186/s12966-021-01080-4

**Published:** 2021-01-22

**Authors:** Cassandra Lane, Sam McCrabb, Nicole Nathan, Patti-Jean Naylor, Adrian Bauman, Andrew Milat, Melanie Lum, Rachel Sutherland, Judith Byaruhanga, Luke Wolfenden

**Affiliations:** 1 School of Medicine and Public Health, The University of Newcastle, Newcastle, NSW Australia; 2 Hunter New England Population Health, Hunter New England Area Health Service, Newcastle, NSW Australia; 3 Priority Research Centre for Health Behaviour, The University of Newcastle, Newcastle, NSW Australia; 4 Hunter Medical Research Institute, New Lambton Heights, NSW Australia; 5 School of Exercise Science, Physical and Health Education, University of Victoria, Victoria, BC Canada; 6 School of Public Health, University of Sydney, Sydney, NSW Australia

**Keywords:** Physical activity, Scale-up, Scale-up penalty, Adaptations

## Abstract

**Background:**

The ‘scale-up’ of effective physical activity interventions is required if they are to yield improvements in population health. The purpose of this study was to systematically review the effectiveness of community-based physical activity interventions that have been scaled-up. We also sought to explore differences in the effect size of these interventions compared with prior evaluations of their efficacy in more controlled contexts, and describe adaptations that were made to interventions as part of the scale-up process.

**Methods:**

We performed a search of empirical research using six electronic databases, hand searched reference lists and contacted field experts. An intervention was considered ‘scaled-up’ if it had been intentionally delivered on a larger scale (to a greater number of participants, new populations, and/or by means of different delivery systems) than a preceding randomised control trial (‘pre-scale’) in which a significant intervention effect (*p* < 0.05) was reported on any measure of physical activity. Effect size differences between pre-scale and scaled up interventions were quantified ([the effect size reported in the scaled-up study / the effect size reported in the pre-scale-up efficacy trial] × 100) to explore any scale-up ‘penalties’ in intervention effects.

**Results:**

We identified 10 eligible studies. Six scaled-up interventions appeared to achieve significant improvement on at least one measure of physical activity. Six studies included measures of physical activity that were common between pre-scale and scaled-up trials enabling the calculation of an effect size difference (and potential scale-up penalty). Differences in effect size ranged from 132 to 25% (median = 58.8%), suggesting that most scaled-up interventions typically achieve less than 60% of their pre-scale effect size. A variety of adaptations were made for scale-up – the most common being mode of delivery.

**Conclusion:**

The majority of interventions remained effective when delivered at-scale however their effects were markedly lower than reported in pre-scale trials. Adaptations of interventions were common and may have impacted on the effectiveness of interventions delivered at scale. These outcomes provide valuable insight for researchers and public health practitioners interested in the design and scale-up of physical activity interventions, and contribute to the growing evidence base for delivering health promotion interventions at-scale.

**Trial registration:**

PROSPERO CRD42020144842.

**Supplementary Information:**

The online version contains supplementary material available at 10.1186/s12966-021-01080-4.

## Background

Physical inactivity is a priority public health issue due to its high prevalence and contribution to the burden of disease [1]. Although many interventions have been trialled internationally to increase levels of physical activity, few effective interventions are scaled-up to reach broader populations. Scaling up is a deliberate process of taking health interventions that have been proven effective on a small scale and expanding their reach into real world settings [2–4]. The World Health Organization has identified scaling-up physical activity interventions as a priority, as doing so is required if they are to have a benefit at the population level [2].

The effectiveness of physical activity interventions when delivered at-scale is not yet well understood [4]. Most physical activity interventions are trialled under optimal research conditions often using resources, infrastructure and expertise that are not readily available in community or clinical settings [5, 6]. If found effective, it is suggested that these interventions be more broadly disseminated to reach a greater proportion of the population who could potentially benefit. However, it has been hypothesised that when delivered at-scale in more real-world contexts, the effects of interventions may attenuate – a phenomenon known as a “scale-up penalty” [7–9] or “voltage drop” [10]. We are not aware of any previous reviews characterising the effects of physical activity interventions that have been scaled-up. However, reviews examining the scale-up of other interventions indicate that an interventions’ effect size at scale-up is generally lower than that reported in pre-scale evaluations, suggesting that scale-up penalties are common [7, 9]. For example, a review of scaled-up developmental preventive interventions with criminal outcomes found that the effects of scaled-up interventions were typically 50–60% (median = 55%) lower [9] than the corresponding pre-scale trial. Similarly, a review of scaled-up obesity interventions found that the effects of scaled-up interventions were typically 75% or less (median = 51.3%) of the effects reported in pre-scale up efficacy trials [7].

Adaptions to interventions are common in the process of scale-up [3, 11, 12] and are often necessary to ensure that interventions can be delivered with the resources of agencies responsible for their implementation. They can also assist the successful expansion of evidence-based practice into larger, uncontrolled environments by improving intervention fit within diverse contexts (e.g., different political climate, economic conditions, public interest) [13]. For example, interventions adapted for culture can be more effective [14] and McCrabb and colleagues found that cultural adaptations were made to deliver health interventions to other population groups [7]. Moreover, adaptations may assist to overcome barriers such as competing priorities within health systems, limited capacities of implementing organisations, and shortages of available resources to facilitate the scale-up process [15]. There are many benefits to adaptation processes for delivering interventions at-scale, however these can also have a detrimental impact on the effects of interventions [7–9]. Research is needed to appraise intervention adaptations and their resulting impact on an interventions’ scalability [16]. This is important for preparing health interventions for scale-up.

Knowledge of whether an intervention deemed effective may have a meaningful population level impact – and whether any adaptations were made that may have impacted this process – is important information for policy makers needing to make decisions regarding the allocation of resources in fiscally constrained environments. The literature regarding the effects and/or adaptations of scaled-up physical activity interventions in community settings has not been subject to a systematic evidence synthesis. We sought to address this evidence gap to contribute to the growing evidence base for delivering health promotion interventions at-scale. Specifically, the objectives of this review were (1) to assess the effects of evidence-based health promotion interventions, delivered in community settings, on measures of physical activity following scale-up; (2) to describe differences in effects of interventions established prior to and following scale-up (scale-up penalty) for comparable measures of physical activity and (3) to describe adaptations made to physical activity interventions occurring as part of the scale-up process.

## Methods

The methods used for this review are based on an existing obesity intervention review by McCrabb and colleagues [7] and were developed using guidance from the *Cochrane Handbook for Systematic Reviews of Interventions* Version 5.1.0 [17]. This review was registered with the international prospective register of systematic reviews (PROSPERO; registration number: CRD42020144842) and conducted as per the Preferred Reporting Items for Systematic Reviews and Meta-Analyses (PRISMA) guidelines.

### Search

We performed a search in July 2019 of six electronic data bases (MEDLINE, EMBASE, PsycINFO, Cochrane Central Register of Controlled Trials [CENTRAL], CINAHL, Education Resources Information Center [ERIC]) using terms from reviews by Milat et al. [18], Reis et al. [13], and Charif et al. [19] relating to scaling-up, physical activity, nutrition, obesity and study design (see Additional file 1). We sought to identify further eligible studies by first reference scanning comprehensive systematic reviews of physical activity interventions and their implementation across a range of settings [20–23] and second, by forward searching via (i) contact with field experts, (ii) contact with authors of interventions included in key systematic reviews, and (iii) email with authors of eligible papers.

### Eligibility criteria

Participants of eligible trials included children, adults and families that have been exposed to a scaled-up health promotion intervention that aimed to improve participants’ physical activity. Studies assessing the intervention were eligible if they were one of a pair of reports (a pre-scale trial and a scaled-up study) which fit the following criteria: (A) the pre-scale report was a randomised controlled trial (RCT) with evidence of efficacy (statistical significance of *p* < 0.05) for at least one trial physical activity outcome measured at the individual level; and (B) the scaled-up study reported on the intentional delivery of the intervention to a greater number of individuals than that of the pre-scale trial. We included interventions that had been vertically scaled (introduced across a whole system at the same time; e.g. a mandated policy or practice) or horizontally scaled (gradually introduced across different sites or groups over time; e.g. phased implementation) [3] to a greater number of participants, new populations, and/or by the means of different delivery systems [24]. For scaled-up studies, we considered studies of any randomised or non-randomised trial design so long as they included at least one measure of physical activity using either objective (e.g., pedometers, accelerometers) or self-reported (e.g., self-reported metabolic equivalent of task [MET]) measures. The report pair (pre-scale and scaled-up) did not have to share a common outcome measure, and studies could be focused on any health-related behaviour, such as obesity or nutrition, provided that they reported on a physical activity outcome. Studies were excluded if (i) participant groups were selected on the basis of pre-existing disease or medical condition, (ii) the primary purpose was replication of the intervention, (iii) they were performed in medical/clinical settings, or (iv) the finding of effectiveness at pre-scale was at the sub-group level.

### Data screening

Title, abstract and full text screening occurred independently by two review authors not blinded to the author or journal information. Google translate was used to assess eligibility of abstracts or articles not published in English. Full texts of manuscripts were obtained for all potentially eligible trials for further examination. Decisions regarding inclusion were made between the review author pair and a third reviewer was consulted to resolve any contrariety.

### Data extraction and management

Two review authors independently extracted data and reached consensus for the following: (i) the characteristics of included studies, such as the country and year of publication, sample, study design, trials measures, and outcomes; (ii) the translation stage of each intervention, categorised using the pathways to scaling-up public health interventions described by Indig et al. [25] (efficacy, effectiveness, implementation, or dissemination); (iii) the nature of any adaptations made for scale-up as characterised using a modified Adaptome model [26]; (iv) information to enable assessment of study quality; and (v) any measures of physical activity reported in a standardised way across both trials (objective and/or subjective measurements).

### Data synthesis

The study characteristics and key findings for measures of physical activity of scaled-up studies are described in Additional file 3. Authors of included studies were emailed where questions of study design, methods, intervention adaptations and/or physical activity outcomes arose during any step of data synthesis. Physical activity outcomes were defined using the inventory of physical activity measures provided by Sylvia and colleagues [27].

We synthesised data in relation to the specific study objectives. First, given the heterogeneity among included studies, we narratively synthesised the effects of interventions on measures of physical activity reported in the included scaled-up studies. Second, we assessed differences in effect size from pre-scale trials to scaled-up studies using the extracted measures reported in a standardised way across both reports. Where a standardised measure was not directly conveyed in each, we sought to calculate a standardised measure for comparison if sufficient data were provided. For example, the physical activity outcome of MET-minutes per week were reported differently across one evaluation pair: the intervention effect was expressed as a regression coefficient in the pre-scale trial [28] and a between-group difference in the scaled-up study [29]. We were able to calculate the between-group difference for the pre-scale trial using the reported pre- and post-intervention MET-minutes per week for intervention and control groups. Thus, we computed a standardised measure for comparison where it was not originally available.

To assess any scale-up penalty, differences in effect size were quantified using the following formula: effect size reported in the scaled-up study divided by the effect size reported in the pre-scale trial and then multiplied by 100. A calculation of 100% indicated that the intervention tested in the scaled-up study had an effect equal to that achieved in the pre-scale trial; values > 100% indicated that the intervention tested in the scaled-up study had a greater effect than it did in the pre-scale trial; and values < 100% indicated that a proportion of the intervention effect was retained following scale-up (a scale-up penalty had occurred). Specific scale-up penalties may be inferred by subtracting the proportion of the intervention effect retained from 100. For example, 25% denoted that the intervention tested in the scaled-up study retained a quarter of the intervention effect following scale-up and had consequently suffered a scale-up penalty of 75%.

Third, we narratively recorded any adaptations made to the intervention based on manuscripts reporting on the pre-scale trial and subsequent scaled-up study. We then searched Google and Google Scholar to identify supplementary material, such as additional studies or published protocols, for additional information where descriptions of interventions within manuscripts were limited, incomplete or unclear.

Adaptations were classified according to the Adaptome model [26] as:

- Service setting adaptations – adaptations to elements of the environment where the intervention delivery takes place, such as the physical location and/or the facilitator.
- Target audience adaptations – adaptations to the population of interest and/or the ‘fit’ for the population of interest, such as expanding eligibility criteria to include students of different grades.
- Mode of delivery adaptations – adaptations to the channel used to deliver the intervention, such as changed frequency of sessions and/or delivering the session in-person versus via the internet.
- Cultural adaptations – adaptions to improve cultural appropriateness of an intervention, such as translating resources into other languages used by populations of interest.
- Other – adaptations that do not fall into the prior categories, such as the addition of a marketing scheme and/or reducing the monetary value of provided resources.

## Results

**Fig. 1:**
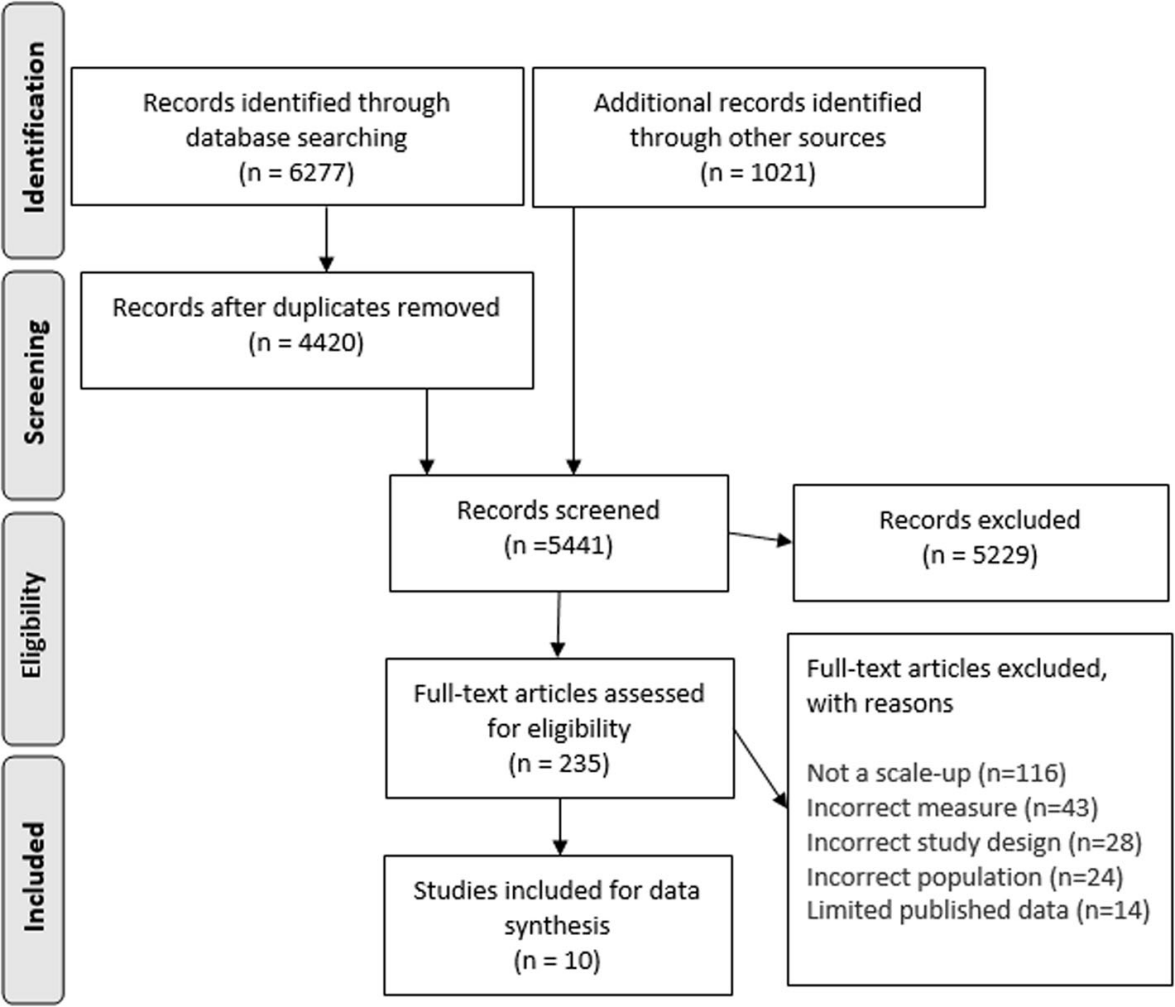
PRISMA (Preferred Reporting Items for Systematic Review and Meta-Analyses) diagram of included studies

Figure 1 displays a PRISMA diagram for this systematic review. The search of databases and additional records identified 5441 titles to screen for eligibility. We contacted corresponding authors of 301 potentially eligible trials, and eight key stakeholders from relevant international organisations. An initial 18 pairs of studies were identified as eligible, six of which were excluded at data extraction for various reasons: the RT for TEENS intervention [30] had been scaled up from an efficacy trial with a statistically significant effect for muscular fitness only, and not for measures of physical activity [31]; the replicated efficacy trial of the CATCH program in Texas was quasi-experimental [32] and there were no evaluations following the original efficacy trial [33] that reported on the intervention with an expanded reach; the pre-scale trial associated with both FUN5! [34] and SPARK [35] were quasi-experimental (one school was purposefully assigned to the control group to account for attrition issues) [35]; the original pre-scale trial of EPODE was a non-RCT design [36]; and it was unclear whether randomisation had been used in the efficacy trial of Exercise Your Options [37]. The Healthy School Start study [38] was excluded after data extraction as the finding of effectiveness in the pre-scale efficacy trial was the result of sub-group analysis [39]. We included a total of 10 scaled-up interventions for review. Table 1 lists the initial interventions tested for efficacy in the pre-scale RCTs and the corresponding scaled-up intervention. Additional file 2 provides an overview of the quality of evidence (internal validity of each study) as assessed using Cochrane’s Risk of Bias tool described in the *Cochrane Handbook for Systematic Reviews of Interventions* [17].

**Table 1:** List of included scaled-up interventions and the corresponding pre-scale efficacy trial intervention

Pre-scale intervention name	Scaled-up intervention name (population, focus)
AS! BC [40]	**AS! BC** *(children, physical activity)* [41, 42]
CHAMPS II [43]	**CHAMPS III** *(older adults, physical activity)* [44]
FFIT [28]	**EuroFIT** *(men, lifestyle)* [29]
MEND [45]	**Go4Fun** *(children, obesity)* [46]
HDHK [47]	**HDHK** *(children, obesity)* [48]
HeLP-her [49]	**HeLP-her** *(women, weight)* [50]
MEND [45]	**MEND 7–13** *(children, obesity)* [51]
SCORES [52]	**SCORES** *(children, physical activity)* [53]
Strong Women-Healthy Hearts [54]	**Strong Women-Healthy Hearts** *(women, lifestyle)* [55]
CLICK-obesity [56]	**YOG-Obesity** *(children, lifestyle)* [57]

### Characteristics of included studies

Additional file 3 provides characteristics of the 10 scaled-up studies included in this review. Three trials focused exclusively on physical activity improvements and the remaining seven trials included physical activity as part of a broader health promotion program for either obesity prevention or a general healthy lifestyle.

Four studies were conducted in Australia [46, 48, 50, 53]; two in the United States [44, 55]; one each from Canada [41, 42], the United Kingdom [51], and China [57]; and the remaining study was conducted across multiple countries (Netherlands, Norway, Portugal, and the United Kingdom) [29].

Target populations of the scaled-up studies varied; two focused on parent-child dyads [46] (one with fathers only [48]); three on primary school students from a variety of grades (grades 3–7) [41, 42, 53, 57]; two on women only (40 years or older [55] and 18–50 years of age [50]), one on men only (30–65 years of age) [29], and the last on older adults (65 years or older) [44].

Four scaled-up studies used a cluster RCT design [41, 42, 50, 53, 57], two used a RCT with participants randomised at the individual level [29, 48], three used a pre-and-post, non-controlled design [44, 46, 55], and one study used an intervention evaluation [51]. Of the scaled-up intervention studies, eight were classified as dissemination efforts [41, 42, 44, 46, 48, 51, 53, 55, 57], and two as effectiveness [29, 50].

### The effectiveness of scaled-up interventions in improving physical activity

Overall, the majority of interventions (6/10) significantly improved at least one measure of physical activity when scaled-up [29, 46, 48, 53, 55, 57]. Four of these findings were from controlled trials randomised at the individual or cluster level. Compared to controls, objectively assessed steps-per-day significantly increased by both children and fathers in the HDHK RCT [48] as well as participants in the EuroFIT RCT [29]. The SCORES cluster RCT [53] showed no significant intervention effect on the primary outcome of students’ total daily minutes of moderate vigorous physical activity (MVPA), however improvements were found with other measures of student physical activity including overall daily vigorous activity, school-day MVPA and school-day vigorous activity. The YOG-Obesity cluster RCT [57] also found an intervention effect for students’ subjectively assessed MVPA. Differently, no significant improvements were found in the HeLP-her cluster RCT [50] for women’s self-reported physical activity (Additional file 3).

The other two scaled-up evaluations that found improvements in physical activity used pre- and post- evaluations. Following the intervention, children in Go4Fun [46] increased the number of days/week they spent in ≥1 h of physical activity and improved their cardiovascular fitness levels while women in StrongWomen-Healthy Hearts [55] increased their mean MET-minutes per week (Additional file 3). Differently, self-reported levels of physical activity by participants in CHAMPS [44] did not significantly improve from pre- to post-intervention (Additional file 3).

The physical activity impacts of the remaining two scaled-up evaluations included in this review are unknown: AS! BC [41, 42] reported on intermediate measures of physical activity (teachers delivered minutes of physical activity) not on the direct physical activity outcomes of participants [41, 42], and MEND 7–13 focused on BMI and omitted measures of physical activity [51] (Additional file 3).

### The effect size difference from pre-scale to scaled-up (scale-up penalty)

Seven studies included at least one standardised measure of physical activity – or sufficient data for our review team to calculate a standardised measure – in both the pre-scale efficacy trial and scaled-up study. The first two columns of Table 2 displays the measures of physical activity common to both reports and column three reports the corresponding proportion of the efficacy trial effect size achieved in the scaled-up trial.

**Table 2:** Effect size difference calculated using measures of physical activity common to both pre-scale trial and scaled-up study

	Pre-Scale RCT	Scaled-up Study	Proportion (%) of the Efficacy Trial Effect Size Achieved in the Scaled-up Study
CHAMPS	RCT Change in intervention group at 12 month follow-up **Weekly caloric expenditure in PA (kcal/week)** + 687, *P* < .001	Pre-post non controlled design Change in intervention group at 6 month follow-up **Weekly caloric expenditure in PA (kcal/week)** + 213, *P* = 0.10	31.0
EuroFIT	RCT Adjusted b/n group difference at 12 weeks and 12 months follow-up **Total MET-min/week** 12 weeks: 1485 12 months: 844	RCT Adjusted b/n group difference at 12 weeks and 12 months follow-up **Total MET-min/week** 12 weeks: 1020, *P* < 0.001 12 months: 894, P < 0.001	68.7 105.9
Go4Fun	RCT Within-subjects change at 6 month follow-up **Recovery heart rate (beats min-1)** − 17.9, *P* < 0.0001	Pre-post non controlled design Within-subjects change post-intervention (10-week follow-up) **Recovery heart rate (beats min − 1)** − 4.64, *P* = 0.002	25.9
HDHK	RCT Mean difference b/n groups at 3 month follow-up **Mean steps per day for fathers** 2139 **Mean steps per day for children** 1228	RCT Mean difference b/n groups at 14 weeks **Mean steps per day for fathers** 1258, *P* = 0.04 **Mean steps per day for children** 1625, *P* = 0.01	58.8 132.3
HeLP-her	RCTA djusted between group difference at 12 month follow-up **Activity self-management score** 0.24, *P* < 0.001	RCT Adjusted between group difference at 12 month follow-up **Activity self-management score **0.06	25.0
SCORES	RCT Adjusted b/n group difference at 6 month follow-up **Total daily MVPA (minutes)** 5.7, *P* = 0.194 **Within-school MVPA (minutes)** 4.1, *P* = 0.211	RCT Adjusted b/n group difference at 6 month follow-up **Total daily MVPA (minutes)** 1.96, *P* = 0.48 **Within-school MVPA (minutes)** 2.90, *P* = 0.05	34.4 70.7

Differences in effect size (i.e., the proportion of efficacy) from pre-scale to scaled-up trials varied. Six trial pairs provided a total of nine comparable measures of physical activity, with differences in effect ranging from 132 to 25% (median = 58.8%; Table 2). Two measures from separate RCTs reported larger effect sizes at follow-up: EuroFIT [29] retained 105.9% of men’s total MET-min/week and HDHK [48] retained 132.3% of children’s mean steps per day. Across all six trial pairs however, the effect size on a measure of physical activity was lower, ranging from 29 to 75% (median = 65.6%) of the effect reported in the pre-scale trial and so representing a scale-up penalty.

### Adaptations occurring for scale-up

Table 3 displays the categories of adaptations that were reported as part of the scale-up process for each intervention. The most commonly reported adaptation among interventions was “mode of delivery”, with all but one intervention reporting at least one adaptation in this category. An example of an adaption of mode of delivery was the addition of a novel technology worn by participants’ in EuroFIT as a means of self-monitoring physical activity [29]. The second most common was “other” (8/10) which included any adaptations that did not fall under the Adaptome [26] categories; for example, the equipment packs provided to SCORES intervention schools were cost-reduced by AUD $820 each [53] and the AS! BC intervention added a healthy eating component [41, 42]. The next most common was “service setting” (7/10), for example the HDHK workshops were conducted at local schools as opposed to the University [48]. Within the remaining categories, four of the 10 intervention trials reported relevant adaptations that fell within “target audience” (e.g., the YOG-Obesity program included an additional three grades of students [57]) and three that fell within “cultural” (e.g., CHAMPS III materials were translated into Spanish [44]). A synopsis of each pre-scale intervention and details of adaptations made for the subsequent scaled-up variation is provided in Additional file 4.

**Table 3:** Adaptations made to physical activity interventions for scale-up based on the Adaptome model [26]

	Mode of Delivery	Service Setting	Target Audience	Cultural	Other
*AS! BC*	X	X	X		X
*CHAMPS*	X	X		X	
*EuroFIT*	X			X	X
*Go4Fun*	X	X		X	X
*HDHK*	X	X			
*HeLP-her*	X	X	X		X
*MEND 7–13*					X
*SCORES*	X	X	X		X
*Strong Women …*	X	X			X
*YOG-Obesity*	X		X		X

## Discussion

This review answers important questions to assess the potential public health benefits of physical activity interventions delivered at-scale. We found that interventions identified as efficacious in controlled research conditions often remained so when scaled-up, although they typically are able to achieve a fraction of the effect sizes reported in pre-scale efficacy trials. With the exception of two improved physical activity outcomes, the effects of scaled-up interventions were lower than at pre-scale (median of 58.8%), suggesting that over half of the effect size might be lost following scale-up. The review also identified that intervention adaptations were common as part of the scale-up process, particularly those made to an interventions’ mode of delivery.

Broadly, the findings of this review are consistent with that of other research regarding the effects of interventions when scaled-up and the extent of the scale-up penalty. Systematic reviews of scaled-up preventive criminological interventions (1) and obesity interventions (2) have similarly found that interventions delivered at scale produce statistically significant effects, but that these effects are often heavily discounted relative to pre-scale evaluations – retaining a median effect size of 55 and 51.3%, respectively. While systematic review evidence suggests that any increased bout of physical activity is associated with improved health outcomes [58], reductions in effects at scale are important considerations for health policy makers given the size of investments required to scale-up interventions. This may be particularly the case among physical activity interventions where effect sizes reported among reviews of pre-scale efficacy trials are modest and further reductions in effect may yield marginal benefits to community health [5]. Nonetheless, this review identified two trials in which greater effect sizes were reported at scale. Further understanding of the types of interventions, delivery systems and context in which improvements in effects can be achieved represents an important area for future health research in order to maximise the potential impact of scaling physical activity interventions.

Consistent with the review by McCrabb and colleagues (2), we found that the most frequently reported adaptations were categorised as “mode of delivery”. Similarly, a 2018 systematic review of 42 evidence-based public health interventions from across the globe (3) found that all of the interventions made “content” adaptations that met the definition for “mode of delivery” used in this review. There are numerous delivery modalities available for health interventions, the choice of which varies by factors such as cost, population reach, and fit with the delivery setting [59]. Modes of delivery used within controlled research conditions are often researcher intensive and costly. Such adaptations are likely necessary to make wide scale implementation of these interventions more achievable – that is, to reach a greater proportion of target populations at a reasonable cost [4, 60]. This might explain the common occurrence of these adaptations for disseminating public health interventions broadly.

Interestingly, some scaled-up reports including EuroFIT [29], HDHK [48], Go4Fun [46] and SCORES [53] explicitly stated that the intervention was designed with the intent to be scaled-up. These interventions tended to report lower penalties than other trials [29, 48, 53]. The development of health interventions with early consideration of scale-up is important [61] and may assist to preserve the effect size. Indeed assessing the effects of interventions using modalities suitable for delivery at-scale and under more naturalistic research conditions might provide better insight for policy makers regarding the effects of these interventions when scaled in the real world, and reduce the magnitude of any scale-up penalty. Such ‘practice-based evidence’ could be generated through quality evaluations undertaken as part of government investments in physical activity program development and delivery. While most government funded health promotion programs are not evaluated [62], innovative and successful models of how researchers can work with and support routine quality pragmatic program evaluations are emerging [63]. There are a number of frameworks [2, 3, 64] and scalability assessment tools (4) available to assist with the design and scale-up of health interventions to support this work. Furthermore, the Pragmatic Explanatory Continuum Indicator Summary Tool (PRECIS-2) [65] describes a range of pragmatic research trial design characteristics that may enhance the applicability of research findings to real world contexts and was designed to help match research design decisions to how the trial results are intended to be used. The more widespread application of such tools in the conduct of physical activity intervention trials appears warranted to facilitate their scale-up.

A number of limitations of this review are worth considering. We used relatively few search terms relating to scale-up and, despite the support of a robust search strategy, we may have missed potentially eligible studies that used comparable terms such as adoption or diffusion. We did not include those interventions which had been scaled-up without an initial RCT nor those without a report of its intentional delivery on a larger scale since an original efficacy trial. For example, although the CATCH intervention is a well-known scaled-up physical activity intervention, there were no eligible scaled-up reports available in the literature. More information regarding other scaled-up physical activity interventions that did not meet our eligibility criteria can be found in the 2018 systematic review by Reis and colleagues (6).

The restriction of this review to interventions that had been evaluated pre-scale via randomised trial may have also excluded other interventions that are not amenable to testing via randomised designs, such as those where changes to systems are undertaken as part of the intervention. Similarly, we identified interventions as effective based on findings of statistical significance (*p* < 0.05). Statistical significance, however, is not a measure of the clinical or public health meaningfulness of improvements in physical activity. In light of these limitations, the review may have omitted interventions that may yield meaningful improvements in physical activity, and therefore have considerable scale-up potential. End-users should be mindful of both the size of the effect and the certainty of the estimate when appraising the benefits of physical activity interventions when scaled-up.

A further limitation is the exclusion of intermediate measures of physical activity (i.e., measures that correspond to an increase in physical activity) from data synthesis. For example, an increase in minutes of physical activity delivered by teachers in AS! BC (7). A broader inclusion criteria may have enabled capture of a greater pool of studies and yielded greater insights into the scale-up process.

Every effort was taken to standardize assessments of pre- and post-scale up effects, for example via comparing the same measure, instrument and pre-post or between group comparisons between pre-scale and scale-up evaluations. As such, we anticipated that differences between effects of interventions were likely due to the process of scaling up, be they related to adaptations to the intervention, its implementation, changes to the population groups (e.g., baseline activity levels) or other contextual factors. Such assertions still require further research to establish important effect modifiers. Further, other methodological differences such as seasonal variations in the assessment period, or the methods employed in statistical analyses may contribute to differences in the estimates of effects between pre- and post-scale reports.

Finally, we were unable to quantify the effect size difference (and potential scale-up penalty) for some interventions. Where we were able to perform this calculation, there were some instances in which the study design and/or primary measure used in the scaled-up evaluation did not match that of its corresponding pre-scale RCT [44, 46]. As interventions move through stages of the research process – from efficacy, replication and effectiveness trials to then test dissemination, implementation and scale-up strategies – the need for assessment of their effects on individual physical activity behaviour (particularly assessed in the same way as efficacy trials) becomes less salient, and arguably unnecessary [66, 67]. Nonetheless, the effects of scaled-up interventions which did not include measures of physical activity in this review, and whether a scale-up penalty has been incurred, remains unknown. The science of scale-up is a nascent field of research; as the evidence base grows so will the opportunity to address several of these limitations.

## Conclusions

Effective public health interventions, including those with an impact on physical activity levels, must be scaled-up to achieve population-wide health improvements [3, 4, 13, 25, 60, 64]. While even small improvements in physical activity can have a positive health impact, the effects of physical activity interventions are typically much smaller than reported in pre-scale efficacy trials. Appraising the extent to which reductions in effect may occur will be important for policy makers and practitioners to assess the likely population health benefits of investment in scale-up.

## Supplementary Information

**Additional file 1.**

**Additional file 2.**

**Additional file 3.**

**Additional file 4.**

**Additional file 5.**

## Data Availability

The datasets used for the current study are available from the corresponding author on reasonable request.

## Abbreviations

PRISMA: Preferred Reporting Items for Systematic Reviews and Meta-analysis
RCT: randomised controlled trial
MET: metabolic equivalent of task
MVPA: moderate vigorous physical activity
