# Supplementary material for: How effective are physical activity interventions when they are scaled-up: a systematic review
Source: Int J Behav Nutr Phys Act. 2021 Jan 22;18:16. doi: 10.1186/s12966-021-01080-4 (PMC7821550; doi:10.1186/s12966-021-01080-4)
Supplement: Supplementary file 1 — Additional file 1. [file 12966_2021_1080_MOESM1_ESM.docx]

Appendix A – Search strategy

| Search topic | Included search terms |
| --- | --- |
| Scaled-up interventions | Scaling-up OR scalability(1)  ("scaling-up" or "scaled-up" or "scale up" or "up-scaling" or "upscaling").ti,ab.  (scalability or scalable or "at scale").ti,ab.  (spread adj5 (innovation* OR intervention* OR technolog* OR practice OR care)).ti,ab.  ((bring* or brought or taking or take* or increas* or going or implement*) adj5 scale)).ti,ab.(2)  scaled-up OR scale-up OR scaling-up OR scalability OR scalable OR reach OR expanding OR expandable OR expandability OR institutionalization OR institutionalisation OR roll-out OR rolling-out OR dissemination OR disseminating(3) |
| Physical activity | 21. exp exercise/  22. physical inactivity.mp.  23. physical activity.mp.  24. exp motor activity/  25. (physical education and training).mp.  26. exp "Physical Education and Training"/  27. exp physical fitness/  28. sedentary.ab. or sedentary.ti.  29. exp life style/  30. exp leisure activities/  31. exp walking/  32. exp sports/  33. exp dancing/  34. dancing.mp.  35. exp exercise therapy/  36. (exercise$ adj aerobic$).tw.  37. (physical$ adj5 (fit$ or train$ or activ$ or endur$)).tw.  38. (exercis$ adj5 (train$ or physical$ or activ$)).tw.  39. sport$.tw.  40. walk$.tw.  41. cycle$.tw.  42. (("lifestyle" or life-style) adj5 activ$).tw.  43. (("lifestyle" or life-style) adj5 physical$).tw.  44. or/21-43(4)  10. exp Exercise/  11. physical inactivity.mp. 12. physical activity.mp. or Physical Activity/ 13. Motor Activity.mp. 14. (physical education and training).mp. 15. Physical Education/ 16. Physical Fitness/ 17. sedentary.mp. 18. exp Lifestyle/ 19. leisure time/ or recreation/ 20. exp Sports/ 21. Dance/ 22. (exercise* adj2 aerobic*).mp. 23. sport*.mp. 24. ((life style or lifestyle) adj5 activ*).mp. 25. (dance* or dancing).mp. 26. or 11 or 12 or 13 or 14 or 15 or 16 or 17 or 18 or 19 or 20 or 21 or 22 or 23 or 24 or 25(5)  physical activity OR physically active OR physical inactivity OR physically inactive OR fitness OR exercis* OR sport* OR walk OR walking OR sedentary OR sitting OR television OR TV OR screen time OR screen-time OR active transport* OR active transit OR active travel OR commut* OR active commuting OR bicycle OR bicycling OR bike OR biking OR active living OR active-living(3)  MeSH terms  Human Activities [I03]  Exercise [I03.305](6) |
| Nutrition | 27. exp Nutrition/ 28. nutrition*.mp. 29. (health* adj2 eat*).mp. 30. Child Nutrition Sciences/ 31. Fruit/ or fruit*.mp. 32. Vegetables/ or vegetable*.mp. 33. canteen*.mp. 34. Food Services/ 35. menu.mp. 36. (calorie or calories or kilojoule*).mp. 37. Energy Intake/ 38. energy density.mp. 39. Eating/ 40. Feeding Behavior/ or feeding behaviour.mp. 41. nutritionary intake.mp. 42. Food Habits/ 43. Food/ 44. Carbonated Beverages/ or soft drink*.mp. 45. soda.mp. 46. sweetened drink*.mp. 47. Nutritionary Fats/ 48. confectionary.mp. 49. (school adj2 (lunch* or meal*)).mp. 50. Menu Planning/ 51. feeding program*.mp. 52. food program*.mp. 53. (nutrition* adj2 program*).mp. 54. cafeteria*.mp. 55. Nutritional Status/ 56. 27 or 28 or 29 or 30 or 31 or 32 or 33 or 34 or 35 or 36 or 37 or 38 or 39 or 40 or 41 or 42 or 43 or 44 or 45 or 46 or 47 or 48 or 49 or 50 or 51 or 52 or 53 or 54 or 55 57. 9 or 26 or 56(5)  MeSH terms  Healthy Nutrition [F01.829.458.205.500]  Nutrition [G07.203.650.240](6) |
| Obesity | #1 MeSH descriptor Overweight explode all trees  #2 MeSH descriptor Body Weight, this term only  #3 (obes* or overweight or over-weight)  #4 MeSH descriptor Body Weight Changes explode all trees  #5 (weight near/2 (loss or lost or losing or reduc*))  #6 (weight near/2 (gain* or increas*))  #7 MeSH descriptor Body Fat Distribution explode all trees  #8 MeSH descriptor Body Mass Index explode all trees  #9 MeSH descriptor Skinfold Thickness explode all trees  #10 MeSH descriptor Waist-Hip Ratio explode all trees  #11 ("body weigh*" or bodyweigh* or "body mass*" or bodymass or "body fat*" or bodyfat*)  #12 MeSH descriptor Overnutrition, this term only  #13 (overeat* or over-eat* or overnourish* or over-nourish* or overnutrit* or over-nutrit*)  #14 (#1 OR #2 OR #3 OR #4 OR #5 OR #6 OR #7 OR #8 OR #9 OR #10 OR #11 OR #12 OR #13)(7)  1. exp Obesity/  2. exp Weight Gain/  3. exp Weight Loss/  4. obes$.af.  5. (weight gain or weight loss).af.  6. (overweight or over weight or overeat$ or over eat$).af.  7. weight change$.af.  8. ((bmi or body mass index) adj2 (gain or loss or change)).af.  9. or/1-8(8)  1. exp obesity/  2. Weight Gain/  3. exp Weight Loss/  4. obes*.mp.  5. (weight gain or weight loss).mp.  6. (overweight or over weight or overeat* or over eat*).mp.  7. weight change*.mp.  8. ((bmi or body mass index) adj2 (gain or loss or change)).mp.  9. 1 or 2 or 3 or 4 or 5 or 6 or 7 or 8(5) |
| Study design | 71. randomized controlled trial.pt.  72. controlled clinical trial.pt.  73. randomized.ab.  74. randomised.ab.  75. clinical trials as topic.sh.  76. randomly.ab.  77. trial.ti.  78. doubleblind.ab.  79. singleblind.ab.  80. experiment*.mp.  81. (pretest or pre test).mp.  82. (posttest or post test).mp.  83. (pre post or prepost).mp.  84. Before after.mp.  85. (Quasi-randomised or quasi-randomized or  quasi-randomized or quazi-randomised).mp.  86. stepped wedge.mp.  87. Preference trial.mp.  88. Comprehensive cohort.mp.  89. Natural experiment.mp.  90. (Quasi experiment or quazi experiments).mp.  91. (Randomised encouragement trial or  randomized encouragement trial).mp.  92. (Staggered enrolment trial or staggered enrollment trial).mp.  93. (Nonrandomised or non randomised or nonrandomized or non randomized).mp.  94. Interrupted time series.mp.  95. (Time series and trial).mp.  96. Multiple baseline.mp.  97. Regression discontinuity.mp.  Or/17-97(9) |

**References**

1. Milat AJ, Bauman A, Redman S. Narrative review of models and success factors for scaling up public health interventions. Implementation Science. 2015;10(1):113.

2. Ben Charif A, Zomahoun HTV, LeBlanc A, Langlois L, Wolfenden L, Yoong SL, et al. Effective strategies for scaling up evidence-based practices in primary care: a systematic review. Implementation Science. 2017;12(1):139.

3. Reis RS, Salvo D, Ogilvie D, Lambert EV, Goenka S, Brownson RC, et al. Scaling up physical activity interventions worldwide: stepping up to larger and smarter approaches to get people moving. The Lancet. 2016;388(10051):1337-48.

4. Dobbins M, Husson H, DeCorby K, LaRocca RL. School-based physical activity programs for promoting physical activity and fitness in children and adolescents aged 6 to 18. Cochrane Database of Systematic Reviews. 2013(2).

5. Wolfenden L, Jones J, Williams CM, Finch M, Wyse RJ, Kingsland M, et al. Strategies to improve the implementation of healthy eating, physical activity and obesity prevention policies, practices or programmes within childcare services. The Cochrane database of systematic reviews. 2016;10:Cd011779.

6. Medicine USNLo. Medical Subject Headings 2018 [Available from: <https://www.nlm.nih.gov/mesh/filelist.html>.

7. Martin A, Booth JN, Laird Y, Sproule J, Reilly JJ, Saunders DH. Physical activity, diet and other behavioural interventions for improving cognition and school achievement in children and adolescents with obesity or overweight. Cochrane Database of Systematic Reviews. 2018(3).

8. Waters E, de Silva-Sanigorski A, Burford BJ, Brown T, Campbell KJ, Gao Y, et al. Interventions for preventing obesity in children. Cochrane Database of Systematic Reviews. 2011(12).

9. Kingsland M, Wiggers J, Wolfenden L. Interventions in sports settings to reduce alcohol consumption and alcohol-related harm: a systematic review protocol. BMJ open. 2012;2(2):e000645.
