# Supplementary material for: How effective are physical activity interventions when they are scaled-up: a systematic review
Source: Int J Behav Nutr Phys Act. 2021 Jan 22;18:16. doi: 10.1186/s12966-021-01080-4 (PMC7821550; doi:10.1186/s12966-021-01080-4)
Supplement: Supplementary file 2 — Additional file 2. [file 12966_2021_1080_MOESM2_ESM.docx]

Appendix B - Risk of Bias

Two reviewers independently assessed risk of bias (RoB) associated with studies of scaled-up interventions using Cochrane’s RoB tool described in the *Cochrane Handbook for Systematic Reviews of Interventions* (1)*,* including extended criteria for cluster RCTs and non-randomised trials. The Cochrane RoB tool guides the assessment of various aspects of trial design, conduct and reporting of any quantitative study. Assessors judged RoB for each scaled-up study as ‘Low’, ‘High’ or ‘Unclear’ using targeted questions within five domains of bias: selection, performance, attrition, reporting, and other (extended criteria). Extended criteria for cluster RCTs included recruitment to cluster, baseline imbalance, loss of cluster, incorrect analysis, contamination and compatibility with individually randomised RCTs. Extended criteria for non-randomised trials included potential confounding.

As assessed by the Cochrane RoB tool, two studies were found to be low risk of bias, with the remaining eight studies assessed as high risk of bias. The most consistently low risk of bias ratings across studies related to incomplete outcome data (seven of 10 studies) and selective outcome reporting (six of nine studies). In regards to cluster RCTs, all five studies assessed achieved low risk of bias for recruitment to cluster, loss of cluster and incorrect cluster. Domains with consistently high risk of bias ratings were blinding of outcome assessment (seven of nine studies) and blinding of participants and personnel (five of 10 studies).

Table 3 - Risk of Bias Assessment

|  |  |  | Random sequence generation | Allocation concealment | Blinding of participants and personnel | Blinding of outcome assessment | Incomplete outcome data | Selective outcome reporting | Recruitment to Cluster | Baseline imbalance | Loss of cluster | Incorrect analysis | Compatibility w/ individually RCTs | Potential Confounding |
| --- | --- | --- | --- | --- | --- | --- | --- | --- | --- | --- | --- | --- | --- | --- |
| AS! BC | | | + | + | ∆ | ∆ | ? | + | + | ? | + | + | ? | / |
| CHAMPS III | | | ∆ | ∆ | ∆ | ∆ | + | ? | / | / | / | / | / | ∆ |
| EuroFIT | | | + | + | + | + | + | + | / | / | / | / | / | / |
| Go4Fun | | | ∆ | ∆ | ∆ | ∆ | ∆ | ? | / | / | / | / | / | ∆ |
| HDHK | | | + | + | + | ∆ | + | + | / | / | / | / | / | / |
| HeLP-her | | | + | + | + | ∆ | + | + | + | + | + | + | ? | / |
| MEND 7-13 | | | ∆ | ∆ | ∆ | / | + | / | / | / | / | / | / | ? |
| SCORES | | | + | + | + | + | + | + | + | + | + | + | ? | / |
| StrongWomen… | | | ∆ | ∆ | ∆ | ∆ | ∆ | ∆ | / | / | / | / | / | ∆ |
| YOG-Obesity | | | + | ? | ? | ∆ | + | + | + | + | + | + | ? | / |

| Risk of Bias | |
| --- | --- |
| High | ∆ |
| Low | + |
| Unclear | ? |
| N/A | / |

**Reference**

1. Higgins JG, S. Cochrane Handbook for Systematic Reviews of Interventions Version 5.1.0 [updated March 2011]. The Cochrane Collaboration, 2011; 2011.
