# Supplementary material for: How effective are physical activity interventions when they are scaled-up: a systematic review
Source: Int J Behav Nutr Phys Act. 2021 Jan 22;18:16. doi: 10.1186/s12966-021-01080-4 (PMC7821550; doi:10.1186/s12966-021-01080-4)
Supplement: Supplementary file 3 — Additional file 3. [file 12966_2021_1080_MOESM3_ESM.docx]

Appendix C - Description of included scaled-up studies

| Author, Year, Country, Intervention name | Design | Setting | Population | Measure(s) of Physical Activity | Follow-up Time Points | Key Findings (Physical Activity) | Translation Stage |
| --- | --- | --- | --- | --- | --- | --- | --- |
| McKay et al., 2015, Canada ^(1, 2)^  AS!BC | Cluster RCT | Thirty schools from four BC provincial health authorities | n = 1,529  Eligibility: students in grades 4 and 5, 8-12 years of age, attending participating schools | Physical activity by accelerometer | Mid-way (1 yr) and post-intervention (20 mo) | Not available | Dissemination |
| Stewart et al., 2006, USA ^(3)^  CHAMPS III | Pre-post non-controlled | Three San Francisco Bay Area community organisations | n = 321  Eligibility: older adults ≥65 years of age | Estimated weekly caloric expenditure and hours spent in physical activity using self-report questionnaire. | Post-intervention (6 mo) | No significant difference between pre- and post-intervention measures | Dissemination |
| Wyke, 2019 (multiple countries) ^(4)^  EuroFIT | RCT | Fifteen football clubs in the Netherlands, Norway, Portugal and the United Kingdom (England) | n = 1,113  Eligibility: men 30-65 years of age, BMI ≥27 kg/m² | Steps per day using the activPAL activity monitor; MET-minutes per week using the IPAQ; and frequency of physically active choices using the Activity Choice Index | Post-intervention (12 wk) and 12 mo from baseline | Significant increase in step count, MET-minutes/week, and the number of participants meeting recommended levels of physical activity –in favour of the intervention | Effectiveness |
| Hardy, 2015, Australia ^(5)^  Go4Fun | Pre-post non-controlled | 15 local health districts across NSW | n = 2,812  Eligibility: children 6-15 years of age, BMI ≥ 85th percentile and a parent/carer | Physical activity ≥1 h/session (days/week) using parent questionnaires and cardiovascular fitness using validated 3-minute step test with 1-minute recovery heart rate | Intervention completion (10 wk) | Significant improvement in days/week spent in ≥1 hour of physical activity per session and in cardiovascular fitness (heart rate recovery) | Dissemination |
| Morgan, 2014, Australia ^(6)^  Healthy Dads Healthy Kids (HDHK) | RCT | The Hunter region of NSW (Singleton and Maitland) | n = 93 fathers  n = 132 children  Eligibility: overweight and obese fathers of primary-school aged children | Father and Child steps/day by pedometer | 14 wk | Significant increase in mean daily steps taken by fathers and children | Dissemination |
| Lombard, 2016, Australia ^(8)^  HeLP-her | Cluster RCT | 41 rural Australian towns | n= 649 participants  Eligibility: Women 18-50 years of age | Physical activity self-management by self-report and related behaviours using the IPAQ long form | Intervention completion (12 mo) | No significant intervention effect | Effectiveness |
| Fagg, 2014, UK ^(9)^  MEND 7-13 | Intervention evaluation using prospective service level data | All regions of England | n = 13,998 participants  Eligibility: overweight children 7-13 years of age and a parent/carer | None | Intervention completion (10 wk) | n/a | Dissemination |
| Sutherland, 2017, Australia ^(10)^  SCORES | Cluster RCT | 46 low socioeconomic elementary schools in the HNE region of NSW | n = 1,139 students  Eligiblity: students in grades 3-6 from randomly selected intervention schools | Students’ minutes of daily MVPA by accelerometer | 6 mo post randomisation | Significant increase in students’ minutes of overall daily VPA as well as school-day MVPA and  VPA | Dissemination |
| Folta, 2015, USA ^(11)^  StrongWomen-Healthy Hearts | Pre-test-post-test within-participants design | 22 American States | n= 345 participants  Eligibility: women ≥40 years of age, BMI ≥24 kg/m², sedentary | Physical activity using the IPAQ short form and self-reported MET | Intervention completion (12 wk) | Significance increase in mean MET-minutes per week | Dissemination |
| Wang, 2018, China ^(12)^  YOG-Obesity | Cluster RCT | 32 primary schools and 16 junior high schools in Nanjing | n=10, 091 students  Eligibility: students in grades 4 and 7 from participating schools | Student’s time spent in leisure time MVPA during past 7 days using the CPAIQ (Children Physical Activity Item Questionnaire) | Post-intervention (10 mo) | Significant increase in student’s 7-day MVPA in favour of the intervention | Dissemination |
| IPAQ = International Physical Activity Questionnaire  MET = Metabolic Equivalent  MVPA = Moderate Vigorous Physical Activity/ VPA = Vigorous Physical Activity | | | | | | | |

**References**

1. McKay HA, Macdonald HM, Nettlefold L, Masse LC, Day M, Naylor P-J. Action Schools! BC implementation: from efficacy to effectiveness to scale-up. Br J Sports Med. 2015;49(4):210-8.

2. Mâsse LC, McKay H, Valente M, Brant R, Naylor P-J. Physical activity implementation in schools: a 4-year follow-up. American journal of preventive medicine. 2012;43(4):369-77.

3. Stewart AL, Gillis D, Grossman M, Castrillo M, McLellan B, Sperber N, et al. PEER REVIEWED: Diffusing a Research-based Physical Activity Promotion Program for Seniors Into Diverse Communities: CHAMPS III. Preventing chronic disease. 2006;3(2).

4. Wyke S, Bunn C, Andersen E, Silva MN, Van Nassau F, McSkimming P, et al. The effect of a programme to improve men’s sedentary time and physical activity: The European Fans in Training (EuroFIT) randomised controlled trial. PLoS medicine. 2019;16(2):e1002736.

5. Hardy LL, Mihrshahi S, Gale J, Nguyen B, Baur LA, O’Hara BJ. Translational research: are community-based child obesity treatment programs scalable? BMC Public Health. 2015;15(1).

6. Morgan PJ, Collins CE, Plotnikoff RC, Callister R, Burrows T, Fletcher R, et al. The ‘Healthy Dads, Healthy Kids’ community randomized controlled trial: A community-based healthy lifestyle program for fathers and their children. Preventive Medicine. 2014;61:90-9.

7. Nyberg G, Norman Å, Sundblom E, Zeebari Z, Elinder LS. Effectiveness of a universal parental support programme to promote health behaviours and prevent overweight and obesity in 6-year-old children in disadvantaged areas, the Healthy School Start Study II, a cluster-randomised controlled trial. International Journal of Behavioral Nutrition and Physical Activity. 2016;13(1).

8. Lombard C, Harrison C, Kozica S, Zoungas S, Ranasinha S, Teede H. Preventing Weight Gain in Women in Rural Communities: A Cluster Randomised Controlled Trial. PLOS Medicine. 2016;13(1):e1001941.

9. Fagg J, Chadwick P, Cole TJ, Cummins S, Goldstein H, Lewis H, et al. From trial to population: a study of a family-based community intervention for childhood overweight implemented at scale. International Journal of Obesity. 2014;38(10):1343-9.

10. Sutherland RL, Nathan NK, Lubans DR, Cohen K, Davies LJ, Desmet C, et al. An RCT to Facilitate Implementation of School Practices Known to Increase Physical Activity. American journal of preventive medicine. 2017;53(6):818-28.

11. Folta SC, Seguin RA, Chui KKH, Clark V, Corbin MA, Goldberg JP, et al. National Dissemination of StrongWomen–Healthy Hearts: A Community-Based Program to Reduce Risk of Cardiovascular Disease Among Midlife and Older Women. American Journal of Public Health. 2015;105(12):2578-85.

12. Wang Z, Xu F, Ye Q, Tse L, Xue H, Tan Z, et al. Childhood obesity prevention through a community-based cluster randomized controlled physical activity intervention among schools in china: the health legacy project of the 2nd world summer youth olympic Games (YOG-Obesity study). International Journal of Obesity. 2018;42(4):625.
