# Supplementary material for: How effective are physical activity interventions when they are scaled-up: a systematic review
Source: Int J Behav Nutr Phys Act. 2021 Jan 22;18:16. doi: 10.1186/s12966-021-01080-4 (PMC7821550; doi:10.1186/s12966-021-01080-4)
Supplement: Supplementary file 4 — Additional file 4. [file 12966_2021_1080_MOESM4_ESM.docx]

Appendix D – Intervention tested for effectiveness in the pre-scale RCT and any adaptations recorded as part of the scale-up process and resulting in the scaled-up intervention

| Program | Pre-scale intervention description | Adaptations made for scale-up |
| --- | --- | --- |
| AS! BC | A participatory, comprehensive, whole-school approach with the aim to create a customised plan that will provide more opportunities for student physical activity throughout the school day. It involved six action zones: school environment, physical education, family and community, classroom action, school spirit and extracurricular. The primary prescriptive component was the classroom action in which teachers were trained in a half day workshop and then continually supported to provide students with an additional 15 minutes of physical activity per school day in addition to regular PE. Each school had an action team composed of school stakeholders, designated champions within schools, and the AS! BC support team. | Mode of delivery:  Regional trainers, district-level monitoring, school success stories, student leadership, and more than 12 different specialty workshops were added and the communication and marketing strategies expanded.  Service setting:  BC provincial government provided political support and 10 years of sustained investment, and AS! BC became embedded within the Directorate of Agencies for School Health (DASH) BC and managed by a cross-government committee.  Target audience:  Program expanded to encompass Kindergarten to grade 7 (from original focus on grades 4-6).  Other:  A healthy eating component within the AS! BC model was added (including a trained dietician and health promotion specialist for the AS! BC Support Team). |
| CHAMPS III | An inclusive choice-based program founded on social cognitive theory; motivational techniques; and self-efficacy enhancement and readiness to change principles that aimed to increase physical activity. Trained University of California, San Francisco (UCSF) staff delivered the program and assisted participants to incrementally increase participation in moderate intensity physical activity to 30 minutes via individualised programs. The one year program consisted of: an informative meeting, an individual planning session support booklet, 10 monthly group workshops, physical activity diaries, monthly newsletters, functional fitness assessments and regular staff-initiated phone calls from a counsellor. | Mode of delivery:  Program duration decreased to 6 months alongside a decreased number of group workshops and the activity diaries, the personal planning session and ongoing motivational telephone support were removed.  Service setting:  Various program aspects were modified to suit each delivery site (e.g., one site used their own staff for content delivery and one site used exercise demonstrations instead of active participation).  Cultural:  Materials were translated into Spanish. |
| EuroFIT | A gender-sensitised, group-based weight loss and healthy living intervention for male fans in Scottish professional football clubs. The 12-month intervention was conducted in two phases. The first – a 12-week active phase – involved weekly 90-minute classroom and active group sessions delivered by community coaching staff at the club's home stadium. Coaching staff were employed by the local football clubs and received two days of training by the research team. The second – a maintenance phase – involved a six month group reunion and six email prompts sent over the course of nine months. | Mode of delivery:  Novel technologies for self-monitoring and to promote competition were added, and email prompts were removed.  Cultural:  Content was culturally-sensitised for each country of implementation (England, Netherlands, Norway and Portugal) to reflect local physical activity and nutrition norms.  Other:  A consortium was established for implementation, weight loss was removed as the core program target, and sedentary behaviour was added as a key target area. |
| Go4Fun | A multicomponent family-based childhood obesity intervention. The six month intervention consisted of 18, two-hour group sessions delivered in a community setting twice a week over nine weeks, followed by a 12-week free family swim pass at a local community pool. Two MEND leaders and an assistant delivered group sessions which focused on nutrition education, behaviour change and exercise. Eight to 15 parent-child dyads and their siblings composed each group. MEND leaders received four days of training and identical materials including detailed methods for group sessions to ensure standardised delivery. | Mode of delivery:  Facilitator training was reduced from four days in-person to two days in-person plus online.  Service setting:  Management and funding was taken over by the NSW Ministry of Health.  Cultural:  The program was translated to reflect the Australian context.  Other:  Implementation took place progressively by local health services and the intervention was marketed to assist with program enrolment. |
| Healthy Dads Healthy Kids  (HDHK) | A three-month family-based program founded on Social Cognitive Theory and Family Systems Theory. Intervention strategies and alignment with theoretical constructs using the taxonomy of behaviour change strategies identified by Abraham and Michie. The aim was to help fathers lose weight, become healthy role models and promote healthy behaviours to their children. Eight, 75-minute sessions were delivered by two male researchers/qualified teachers with PE expertise. Fathers are joined by their child/children for three sessions focused on fundamental movement skills, rough and tumble play, health-related fitness, and fun and active games. Participants were provided resources such as a physical activity handbook, weight loss handbook and an online component. | Mode of delivery:  The number of sessions including children increased from three to four and the duration of sessions increased from 75 to 90 minutes.  Service setting:  Workshops were conducted at local schools instead of the University and delivered by local PE teachers who underwent program-specific training. |
| HeLP-her | A self-treatment intervention that aimed to prevent weight gain in women with young children. The one year program consisted of: a) one, 60-minute group session during weeks one, two, three and 16 delivered by the same trained facilitator (a nutritionist) at the local primary school, with a focus on behaviour change skills for nutrition and physical activity; b) provision of a pedometer to self-monitor physical activity; and c) follow-up support via personalised monthly text messages starting week four. Participants were encouraged to take part in low intensity voluntary physical activities and regular self-weighing. | Mode of delivery:  The number of group sessions decreased from four to only one, and a program manual (information and activities) and 20 minutes of coaching over the telephone were added.  Service setting:  Facilitators increased from one to multiple individuals and each were required to have a tertiary qualification in health sciences.  Target audience:  The target population changed from women with a child attending primary school to women 18-50 years of age residing near a participating town.  Other:  Theoretical influences of motivational interviewing, a communication plan, and an engagement framework were added. |
| MEND 7-13 | As described for Go4Fun (above). | Other:  MEND Central was established and considered important for national implementation support. |
| SCORES | A 12-month multicomponent intervention founded on the socioecological model with the aim to increase physical activity and improve fundamental movement skills for children attending primary schools in low-income communities. The following intervention components were delivered in three phases: a) student leadership to increase physical activity at lunch and recess, b) teacher professional learning workshops, c) parental engagement, d) researchers meeting with school staff to discuss policy and environment, and e) community links. | Mode of delivery:  Ongoing support (four videos reinforcing teaching principles) and stickers during PE lessons as a student incentive were added; the community links component was removed; schools provided recess and lunchtime physical activity rewards in any way they saw fit (as opposed to the previous program-specific reward booklets); the program duration decreased to six months; teacher professional development was reduced to one 90-min session compared to the half day training or full day training for stage two teachers; and the parent engagement component lessened from four newsletters, a parent evening and homework to only three newsletters.  Service setting:  The program was adopted as part of routine delivery of health promotion services by Hunter New England Population Health (HNEPH).  Target audience:  The program expanded from the original focus on only students in grades three and four to include all students.  Other:  The equipment pack cost was reduced from $1000 to $180 (Australian Dollars). |
| Strong  Women-  Healthy Hearts | A community-based intervention that aimed to reduce the risk of cardiovascular disease in middle age overweight/obese women. The 12-week intervention consisted of 24 group sessions, delivered twice weekly by a program educator who had been recruited by a state-level organisation and trained by research staff. Each one hour session was divided into a 30-minute physical activity component and a 30-minute nutrition component. Study personnel at each site observed one class to assess intervention fidelity. | Mode of delivery:  Educators were trained via a series of seminars on all program aspects as opposed to the previous informal training by research staff, and each classroom curriculum increased to 45 minutes.  Service setting:  Educators were recruited using a different national organisation.  Other:  Nutrition guidelines were formed using the American Heart Association nutrition advice and the 2010 Nutrition Guidelines for Americans instead of general healthy eating practices of an unspecified source. |
| YOG-Obesity | A school-based multi-component lifestyle intervention developed with full consideration of Chinese culture and based on several behavioural theories including the Theory of Triadic Influence (TTI) and the comprehensive School Health Program Model (CSHPM). The program aimed to reduce obesity risk and increase health behaviours and knowledge of students in China. It consisted of the following four components: a) nutrition and physical activity classroom curriculum delivered monthly by classroom teachers during one 30-minute lesson; b) school environment support; c) family involvement (e.g., parent/guardian health class delivered by research team personnel two times a semester at each school); and d) fun program/events. All components were integrated into each school's normal academic routine. | Mode of delivery:  The program expanded (see target audience) and was delivered in junior high schools in addition to primary schools. Three, one-week activities were developed for all intervention students.  Target audience:  The program expanded from the original focus on students in grade 4 to include those in grades 4 through 7.  Other:  An official document asking schools to integrate the program was issued by the Nanjing Municipal Education Department & Health Department, and the Nanjing Centre for Disease Control provided technical support for the intervention programs; the healthy eating component was removed and an emphasis was placed on physical activity. |
